# Progress and challenges in transplantation of human pluripotent stem cell derived cardiomyocytes for cardiac therapy

**DOI:** 10.1038/s44385-025-00048-4

**Published:** 2026-01-07

**Authors:** Jacelyn D. Bain, Ryan W. Barrs, Ying Mei

**Affiliations:** 1 Bioengineering Department, Clemson University, Clemson, SC USA; 2 College of Dental Medicine, Medical University of South Carolina, Charleston, SC USA; 3 Department of Regenerative Medicine and Cell Biology, Medical University of South Carolina, Charleston, SC USA

**Keywords:** Heart failure, Regenerative medicine, Stem-cell biotechnology, Tissue engineering, Preclinical research, Translational research

## Abstract

Myocardial infarction and heart failure remain leading causes of mortality worldwide. Human pluripotent stem cell-derived cardiomyocytes (hPSC-CMs) represent a promising approach to regenerating damaged myocardium and restoring cardiac function. This review highlights advancements in hPSC-CM differentiation, scale-up, and clinical-grade manufacturing; delivery approaches; and insights from preclinical and clinical studies. We also examine mechanisms of repair, key challenges and mitigation strategies, and future directions to advance hPSC-CM therapies toward clinical translation.

## Introduction

Each year, more than 735,000 people in the United States suffer from myocardial infarction (MI), leading to the loss of up to one billion cardiomyocytes in the left ventricle and a substantial decline in cardiac contractile function^1,2^. While revascularization strategies such as percutaneous coronary intervention (PCI) reduce acute mortality, many patients still progress to heart failure (HF) due to the heart’s limited regenerative capacity^3,4^.

Cell therapies have been investigated to address this unmet need. Early trials using non-cardiac cells, including bone marrow-derived mesenchymal stem cells (MSCs), showed only modest functional benefit, largely attributed to paracrine effects rather than true remuscularization^5,6^. These limitations shifted focus towards cardiomyocytes derived from human pluripotent stem cells (hPSC-CMs), from both embryonic stem cells (hESCs) and induced pluripotent stem cells (iPSCs), which have demonstrated robust remuscularization and functional cardiac recovery in preclinical studies^7–13^. With growing evidence from preclinical studies and increasingly efficient protocols for hPSC-CM differentiation, hPSC-CMs have emerged as a leading candidate for next-generation cardiac cell therapy^14,15^.

This review highlights recent advances in manufacturing, including hPSC-CM differentiation, purification, and clinical-scale production; delivery approaches; and insights from preclinical and clinical studies. We also discuss key remaining challenges: cell survival, engraftment arrhythmias (EAs), and immune rejection; and strategies to mitigate them. Finally, we outline future directions for advancing hPSC-CM therapies toward clinical translation.

## Manufacturing of clinical-grade hPSC-CMs

### Differentiation and purification

Primary cardiomyocytes have limited therapeutic potential due to challenges in their isolation and cultivation^16^. The advent of hPSCs enabled the derivation of unlimited numbers of functional human cardiomyocytes for therapeutic applications. Early spontaneous differentiation protocols led to less than 1% hPSC-CMs, insufficient for therapy^17^. Directed differentiation protocols using BMP4 and Activin A improved yields to >30% hPSC-CMs^18,19^. Sequential Wnt/β-catenin activation (days 0-1) followed by inhibition (days 3–5) now routinely yields >90% hPSC-CMs^18–20^.

To further enhance purity, lactate metabolic selection leverages differences in glucose and lactate metabolism between cardiomyocytes and non-cardiomyocytes, resulting in hPSC-CM populations with a purity of up to 99%^15^. Despite these advances, single cell RNA sequencing studies have revealed the heterogeneous nature of hPSC-CMs, composed of subpopulations including atrial-specific cells expressing MYL7 and NPPA; ventricular-specific cells expressing MYL2 and IRX4; and nodal-like cells expressing HCN4, SHOX2, and TBX3^21–24^.

Subtype specific hPSC-CM differentiation protocols have therefore been gaining attention. For example, ventricular specification is promoted by retinoic acid (RA) inhibition or modifying BMP4 and Activin A levels, while atrial specification is enhanced by stimulating the RA pathway^22,23,25,26^. By integrating efficient differentiation protocols, metabolic purification, and subtype-directed approaches, hPSC-CMs can be developed into standardized therapeutic products with defined clinical functionality.

### Clinical-scale production

The loss of 1 billion cardiomyocytes in an MI necessitates efficient and robust methods for hPSC-CM differentiation and expansion. Large scale expansion after differentiation enables a ~250-fold increase in hPSC-CM numbers within 4–5 passages^27^. Efficient cryopreservation further allows pooling of hPSC-CMs from multiple batches to generate the quantities required for therapeutic application^28^. Bioreactors have emerged as a promising approach to increase hPSC-CM yields. For example, stirred-tank reactors generate 1.8*10^6^ hPSC-CMs per mL, achieving ~94% viable cells after cryopreservation, requiring a minimal footprint^29^. Similarly, Chen et al leveraged canonical Wnt signaling in a bioreactor, producing 1.5–2*10^9^ hPSC-CMs per liter with 91–92% purity and 85% recovery post-cryopreservation^30^. Dhahri et al. applied the BMP4-Activin A protocol in a PDMS lined 1 liter roller bottle yielding 1.2*10^8^ mature hPSC-CMs per liter^31^. Together, advances in bioreactor platforms, large-scale expansion, and cryopreservation are enabling the production of clinically relevant numbers of hPSC-CMs for transplantation.

## Transplantation methods

A variety of approaches, including intracoronary, systemic intravenous, and retrograde coronary venous injections, were initially tested for hPSC-CM transplantation into infarcted myocardium. However, these methods resulted in poor cell retention and had limited functional recovery^32^. Consequently, current hPSC-CM delivery strategies have shifted towards intramyocardial injections and epicardial patches, which demonstrate improved engraftment and therapeutic potential.

### Intramyocardial injection

**Fig. 1:**
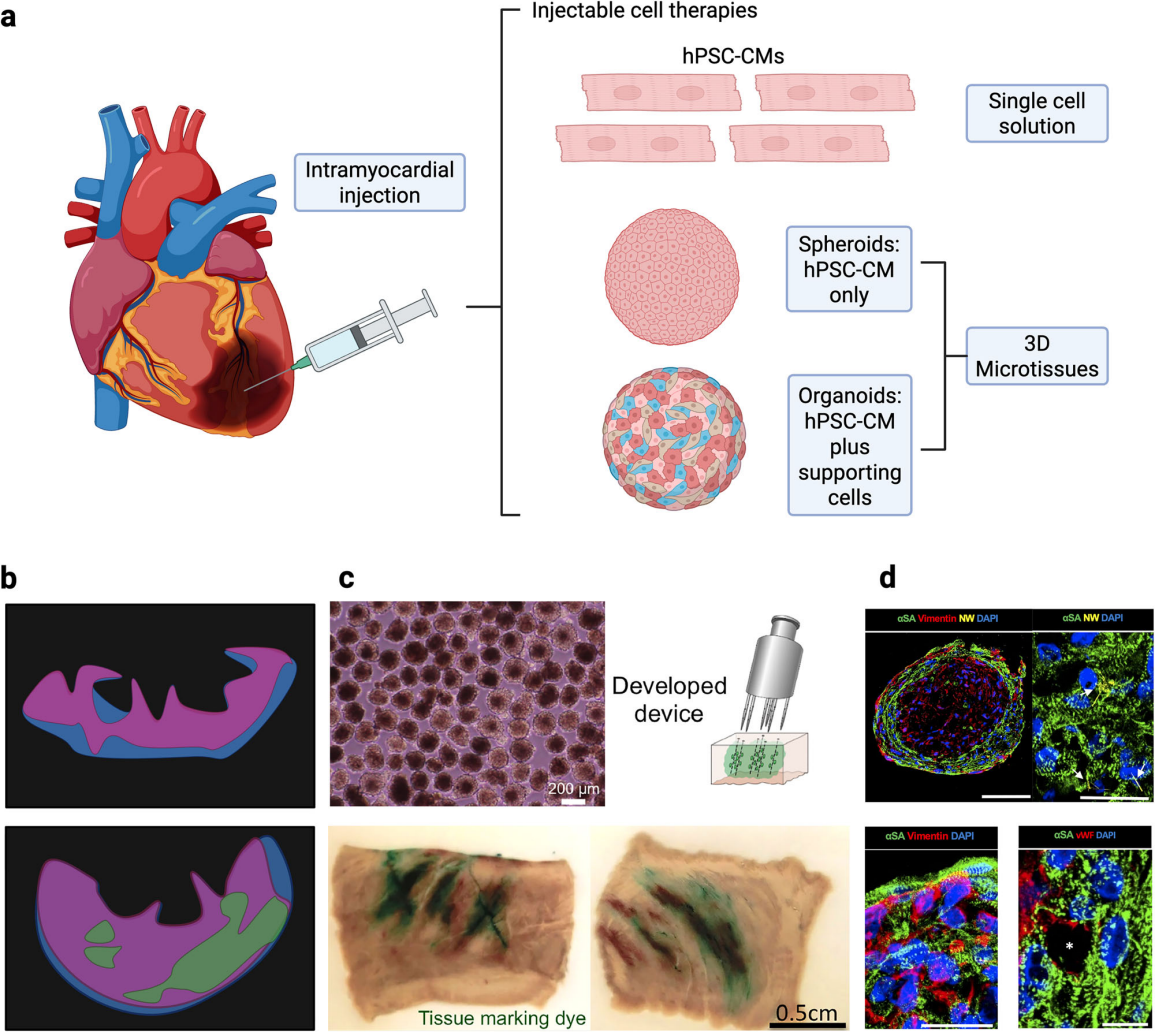
Intramyocardial injections components and key developments. **a** Intramyocardial injections can be single cell solutions or 3D microtissues. **b** Inspired by a figure of a large hPSC-CM engraftment by Liu et al.^41^. (Top) control heart. (Bottom) hPSC-CM treated heart. The blue regions represent collagen 1, the pink is cTnT, and the green is human cTnI. **c** Fukuda et al. specialized delivery system. (Top left) spheroids in phase contrast^42^. (Top right) schematic of spheroid distribution upon injection^42^. (Bottom) distribution of tissue marking dye delivered through the injection device (left) long axis (right) short axis^42^. **d** Nanowired organoid by Tan et al. (Top left) stained for alpha sarcomeric actinin (green), Vimentin (red) nanowires (yellow) and DAPI (blue) (top right) stained for alpha sarcomeric actinin (green), nanowires (yellow) and DAPI (blue) (Bottom left) stained for alpha sarcomeric actinin (green), Vimentin (red) and DAPI (blue) (bottom right) stained for alpha sarcomeric actinin (green), von Willebrand Factor (red) and DAPI (blue)^9^. **c** Reprinted from ref. ^42^ Copyright (2019), with permission from Elsevier. **d** From Tan et al. Nanowired human cardiac organoid transplantation enables highly efficient and effective recovery of infarcted hearts. Science Advances 9, eadf2898 10.1126/sciadv.adf2898. Reprinted with permission from AAAS.

Intramyocardial injections deliver cells directly into the myocardium using syringes or specialized catheters (Fig. 1a). Although overall cell retention is modest (~1–10%), robust engraftments of hPSC-CMs within the infarcted left ventricle of non-human primate (NHP) hearts have been demonstrated (Fig. 1b). For example, Chong et al. reported mean engraftments of 2.1% (0.7–5.3%) of the infarcted region in NHPs^33^. Engrafted hPSC-CMs form electromechanical connections with host cardiomyocytes, contributing to functional recovery in infarcted hearts^33–41^.

Beyond single-cell suspensions, intramyocardial injections have been used to deliver 3D hPSC-CM microtissues (Fig. 1a). Compared to single cell suspensions, microtissues demonstrate improved cell retention, as demonstrated by a study comparing the retention of 20 μm (32.4 ± 10.8%) and 175 μm (48.7 ± 14.3%) fluorescent beads following transplantation^42^. Building on this, Fukuda et al. developed a multicomponent delivery system consisting of a specialized syringe attachment, a gelatin hydrogel, and purified hPSC-CM spheroids^42^ (Fig. 1c). The syringe attachment featured six needles, with multiple holes on the side of each needle, reducing spheroid backflow and dispersing cells more evenly in the myocardium. Delivery of hPSC-CM spheroids using this system improved cardiac function, including increased ejection fraction, in rat and pig heart failure models^10,43^.

3D hPSC-CM microtissues also enable the incorporation of supportive cell types and biomaterials. For example, vascular cells, such as endothelial cells, have been incorporated into 3D microtissues to enhance graft survival and maturation^9,44^ (Fig. 1d). Additional supporting cells, including fibroblasts and pericytes, have been explored to further promote engraftment. For example, Min et al. designed a microtissue system incorporating multiple cell types, cardiac extracellular matrix, and fluid flow to create macroscale tissue aggregates^45^. Delivery of these microtissues into a rat ischemia-reperfusion model improved cardiac function, evidenced by increased left ventricular ejection fraction and fractional shortening.

### Epicardial patches

**Fig. 2:**
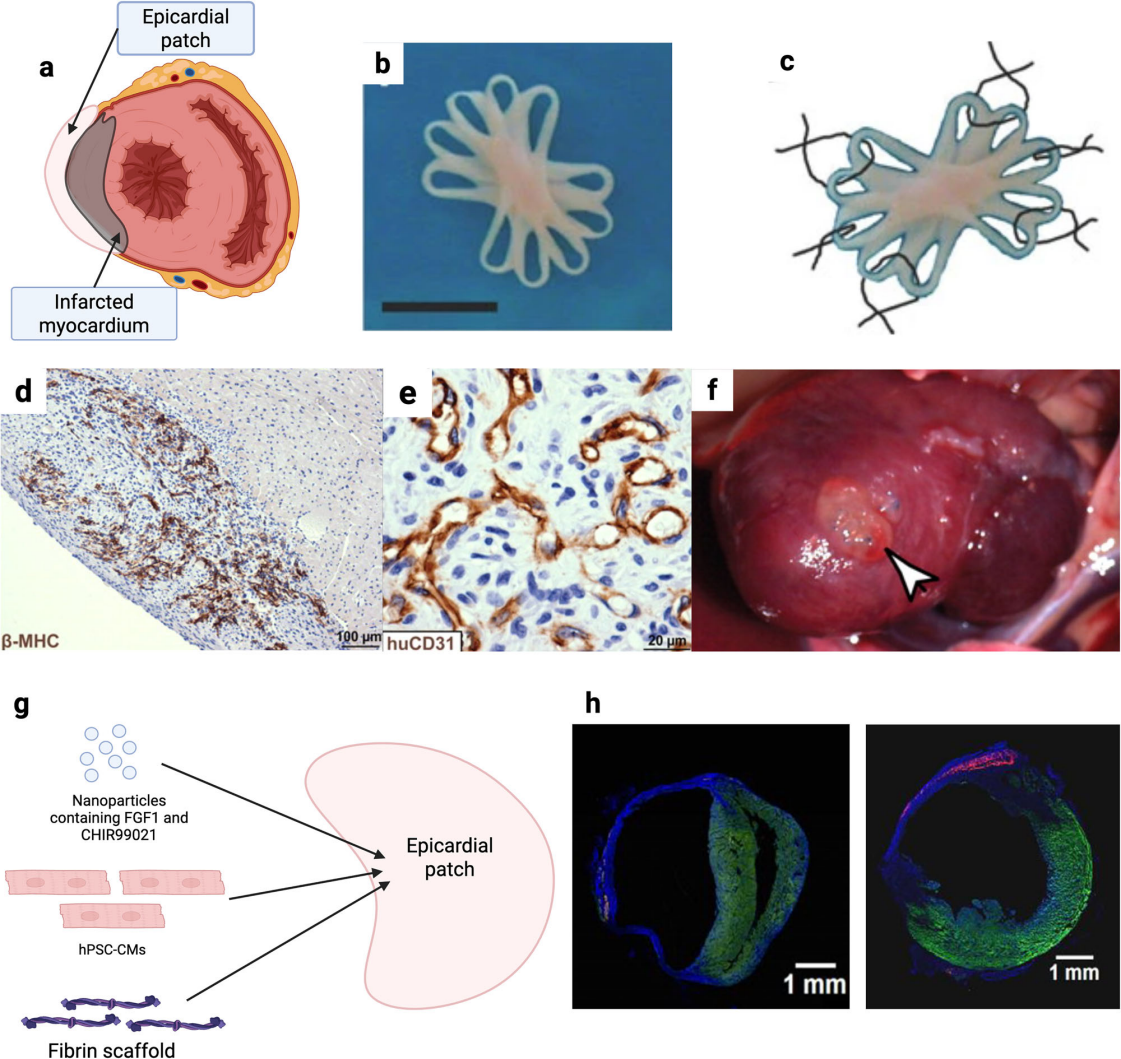
Epicardial patch components and key developments. **a** Epicardial patches are applied to the external layer of the heart. **b**, **c** Scaffolded patch composed of hPSC-CMs and a fibrin scaffold^7^. **b** Multiloop scaffolded patch. **c** Patches are secured to the heart by 6 sutures. **d**–**f** Scaffold free patch with hPSC-CMs + human umbilical vein endothelial cells + fibroblasts^48^. **d** beta myosin heavy chain expression in the patch. **e** Lumen structures form in the patch. **f** Patch attached to the outside of the heart. **g** Schematic of scaffold-based epicardial patch fabrication with hPSC-CMs, biomaterials, and bioactive factors. **h** Patch CMs’ retention (red) in infarcted mice hearts without bioactive factors (left) or with CHIR99021 + FGF1 nanoparticles (right)^80^. **b**, **c** Reproduced from ref. ^7^. **d**–**f** Reproduced from ref. ^48^. With permission from PNAS. **h** Reprinted from ref. ^80^ Copyright (2020), with permission from Elsevier.

Epicardial patches provide another viable strategy to engraft hPSC-CMs onto the surface of infarcted myocardium (Fig. 2a). Zimmermann and Eschenhagen pioneered this approach in 2006 by implanting large engineered cardiac tissues composed of primary rat CMs onto infarcted rat hearts^7^ (Fig. 2b, c) and later demonstrated successful engraftment in a human heart^46^. Compared with intramyocardial injection, epicardial patches offer greater structural support, enhancing hPSC-CM retention (>10%).

There are two main approaches to patch fabrication, scaffold-free and scaffolded. Scaffold free patches rely on self-organized tissue sheets. For example, thermoresponsive poly(N-isopropylacrylamide) has been used to generate sheets of hPSC-CMs that detach from culture substrates at low temperatures and then stacked two to three layers thick^47^. Alternatively, Stevens et al developed scaffold free hPSC-CM patches using suspension culture on a rotating orbital plate, incorporating vascular supporting cells to promote vascularization and improve the functions of the patches^48,49^ (Fig. 2d–f).

Scaffold-based patches embed hPSC-CMs within biomaterials such as fibrin or collagen, often supplemented with bioactive factors (Fig. 2g). In cryoinjury guinea pig models, functional recovery has been demonstrated, although electrical integration between the patch and the host was limited, with 3 out of 10 subjects demonstrating coupling across two studies^12,50^. Importantly, transplantation of a clinically relevant size fibrin-based patch composed of hPSC-CMs, smooth muscle cells (SMCs), and endothelial cells into pig models increased ejection fraction and decreased infarct size^8^. Querdel et al. further demonstrated that patch engraftment and functional recovery was dependent on the dose of cells delivered by the patch^51^.

## Preclinical models of myocardial infarction and heart failure

Rodent models were essential for initial proof-of-concept studies; however their high heart rates (HR) (400 and 600 beats per minute (bpm)) obscure engraftment arrhythmias (EAs). Guinea pigs exhibit action potentials more similar to humans, but their high collateral coronary blood flow makes creating a severe enough infarct by ligation difficult. Pig hearts are anatomically and electrophysiologically similar to humans (HR 80–100 bpm), while non-human primates (HR 120–150 bpm) provide the closest electrophysiological match, enabling more accurate arrhythmia assessment. EAs have been shown to be present in these models, with fatal arrhythmias observed in pig models^52^.

Three main approaches are used to induce myocardial injury. Permanent ligation of coronary arteries, particularly the left anterior descending artery (LAD), models a transmural MI^7,53^. Ischemia reperfusion (IR) models mimic a PCI treated MI, typically requiring 60–180 min of ligation followed by reperfusion^9,13,22,41,54–56^. Cryoinjury models create precise infarct borders, commonly used in guinea pig models^12,38,50^.

The dosing of transplanted hPSC-CMs varies widely between studies and between animal models. The timing of hPSC-CM delivery has been studied across acute^7,12,13,22,38,41,50,57^, subacute^9,39,56^, and chronic phases^54,55^. While most studies focus on acute and subacute treatment phases of MI, increasing attention is shifting toward chronic HF, especially with the initiation of human clinical trials in HF patients^54,58,59^. Table 1 summarizes models, injury induction, cell doses, and endpoints.

**Table 1:** Animal models

Title	Reference	Animal model	Treatment type	Injury model	Treatment delivery timepoint	Experiment final endpoint	Cell species	Cell types included	Culture duration	Scaffold material	hPSC-CMs dose	Total cell dose
Human ES cell derived cardiomyocytes electrically couple and suppress arrhythmias in injured hearts	^38^	Guinea pig	Single cell suspension	Cryoinjury	10 days post injury	28 days post-transplant	Human	hPSC-CM	2–3 weeks	n/a	1*10^8^	1*10^8^
Human embryonic stem cell derived cardiomyocytes regenerate non human primate hearts	^33^	Monkey	Single cell suspension	Ischemia reperfusion (90 min)	14 days post injury	84 days post-transplant	Human	hPSC-CM	16–20 days	n/a	1*10^9^	1*10^9^
Allogenic transplantation of IPS cell derived cardiomyocytes regenerates primate hearts	^91^	Monkey	Single cell suspension	Ischemia reperfusion (3 h)	14 days post injury	84 days post-transplant	Monkey	CMs	17 days	n/a	4*10^8^	4*10^8^
Human embryonic stem cell derived cardiomyocytes restores function in infarcted hearts of non human primates	^41^	Monkey	Single cell suspension	Ischemia reperfusion (3 h)	14 days post injury	12 weeks post-transplant	Human	hPSC-CM	21 days	n/a	7.5*10^8^	7.5*10^8^
Cardiomyocytes derived from human embryotic stem cells in pro-survival factors enhance function of infarcted rat hearts	^56^	Rat	Single cell suspension	Ischemia reperfusion	4 days post injury	4 weeks post-transplant	Human	hPSC-CM	2–3 weeks	n/a	10*10^6^	10*10^6^
Electrical integration of human embryonic stem cell derived cardiomyocytes in a guinea pig chronic infarct model	^59^	Guinea pig	Single cell suspension	Cryoinjury	28 days post injury	4 weeks post-transplant	Human	hPSC-CM	15–17 days	n/a	80*10^6^	80*10^6^
Gene editing to prevent ventricular arrhythmias associated with cardiomyocyte cell therapy	^81^	Pig	Single cell suspension	Healthy	No injury	7 weeks post-transplant	Human	hPSC-CM	18–20 days	n/a	150*10^6^	150*10^6^
Human embryonic stem cell derived cardiomyocytes engraft but do not alter cardiac remodeling after chronic infarction in rats	^54^	Rat	Single cell suspension	Ischemia reperfusion	1 month post injury	3 months post-transplant	Human	hPSC-CM	2–3 weeks	n/a	10*10^6^	10*10^6^
Non contractile stem cell cardiomyocytes preserve post infarction heart function	^65^	Rat	Single cell suspension	Ischemia reperfusion	4 days post injury	84 days post-transplant	Human	hPSC-CM	20 days	n/a	1*10^8^	1*10^8^
Cellular heterogeneity of pluripotent stem cell derived cardiomyocyte grafts is mechanistically linked to treatable arrhythmias	^22^	Pig: landrace	Single cell suspension	Ischemia reperfusion	17 days post injury	28 days post-transplant	Human	hPSC-CM	15 days	n/a	750*10^6^	750*10^6^
Regeneration of nonhuman primate hearts with human induced pluripotent stem cell derived cardiac spheroids	^13^	Monkey	Spheroids	Ischemia reperfusion	14 days post injury	84 days post-transplant	Human	hPSC-CM	2–3 weeks hPSC-CMs	n/a	2*10^7^	2*10^7^
									9 days spheroids	n/a	6*10^7^	6*10^7^
Intramyocardial transplantation of human ips cell derived cardiac spheroids improves cardiac function in heart failure animals	^10^	Pig	Spheroids	Cryoinjury	4 weeks post injury	8 weeks post-transplant	Human	hPSC-CM	2–3 weeks hPSC-CMs	n/a	1*10^8^	1*10^8^
									9 days spheroids			
Spheroids of cardiomyocytes derived from human induced pluripotent stem cells improve recovery from myocardial injury in mice	^53^	Mouse	Spheroids/Patch	LAD ligation	Day of injury	4 weeks post-transplant	Human	hPSC-CM	16 days hPSC-CMs	Fibrin	10*10^5^	10*10^5^
									7 days spheroids			
Implanted human cardiac spheroids electrically couple with infarcted swine myocardium	^92^	Pig	Spheroids	Ischemia reperfusion	0 days post injury	1 week post-transplant	Human	hPSC-CM	12 days	n/a	50*10^6^	50*10^6^
Nanowired human cardiac organoids transplantation enables highly efficient and effective recovery of infarcted hearts	^9^	Rat	Organoids	Ischemia reperfusion	4 days post injury	28 days post-transplant	Human	hPSC-CM (55%), HUVEC (14%), FB (24%), ADSC (7%)	14 days hPSC-CMs	n/a	500*10^3^	~909*10^3^
									4 days organoids			
Engineered heart tissue grafts improve systolic and diastolic function in infarcted rat hearts	^7^	Rat	Patch	Permanent LAD ligation	14 days post injury	28 days post-transplant	Rat	CMs	17–19 days	Collagen	2.5* 10^6^ (*5)	2.5* 10^6^ (*5)
Human engineered heart muscles engraft and survive long term in a rodent myocardial infarction model	^55^	Rat	Patch	Ischemia reperfusion	1 month post injury	28 days post-transplant	Rat	CMs	17–19 days	Collagen 1	2.5*10^6^ (*2)	2.5*10^6^ (*2)
Cardiac repair in guinea pigs with human engineered heart tissue from induced pluripotent stem cells	^12^	Guinea pig	Patch	Cryoinjury	7 days post injury	28 days post-transplant	Human	hPSC-CM	14 days	Fibrin	1*10^7^	1*10^7^
Human engineered heart tissue patches remuscularize the injured heart in a dose-dependent manner	^51^	Guinea pig	Patch	Cryoinjury	7 days post injury	4 weeks post-transplant	Human	hPSC-CM	14 days	Fibrin	4.5-12*10^6^	4.5–12*10^6^
Human ipcs cell derived engineered heart tissue does not affect ventricular arrhythmias in a guinea pig cryoinjury model	^50^	Guinea pig	Patch	Cryoinjury	7 days post injury	28 days post-transplant	Human	hPSC-CM, endothelial cells	14 days	Fibrin	5*10^6^	7*10^6^
Physiological function and transplantation of scaffold free and vascularized human cardiac muscle tissue	^48^	Rat	Patch	Healthy	No injury	7 days post-transplant	Human	hPSC-CM, endothelial cells, fibroblasts	13–15 days hPSC-CMs	n/a	2*10^6^	5*10^6^ (1:1:0.5)
									8–11 days patch			
Enhanced electrical integration of engineered human myocardium via intramyocardial versus epicardial delivery in infarcted rat hearts	^39^	Rat	Single cell suspension	Ischemia reperfusion	4 days post injury	4 weeks post-transplant	Human	hPSC-CM	21–24 days	n/a	10*10^6^	10*10^6^
			Spheroids					hPSC-CM	1 days	n/a	10*10^6^	10*10^6^
			Patch					hPSC-CM	6-7 days	n/a	10*10^6^	10*10^6^
Cardiac repair in a porcine model of acute myocardial infarction with human induced pluripotent stem cell derived cardiovascular cell populations	^57^	Pig	Single cell suspension	Ischemia reperfusion	Day of injury	4 weeks post-transplant	Human	hPSC-CM	2–3 weeks	n/a	6*10^6^	6*10^6^
			Single cell suspension					hPSC-CM, endothelial cells, SMC		n/a	2*10^6^	6*10^6^ (1:1:1)
			Patch					hPSC-CM, endothelial cells, SMC		Fibrin	2*10^6^	6*10^6^ (1:1:1)

Table of important animal model experiments.

*n/a* not applicable.

## Clinical translation

Building on the promising preclinical findings, several human clinical trials have been initiated using single cell suspensions, spheroids, and epicardial patches. Table 2 summarizes ongoing and completed human trials including therapy type, inclusion criteria, and primary outcomes.

**Table 2:** Human clinical trials

Trial title	Treatment type	Status	Estimated enrolled	estimated completion date	NYHA classification	LVEF	Cell type	Delivery method	Primary outcome measures	Ages	ID number
Allogeneic iPSC-derived cardiomyocyte therapy in patients with worsening ischemic heart failure	Cell	Recruiting	32	7/31/2025	Class 3–4	Below 40%	hiPSC-CM	Epicardial injection	Safety, incidence and severity of adverse events	35–75	NCT05566600
Epicardial injection of hiPSC-CMs to treat severe chronic ischemic heart failure	Cell	Recruiting	36	3/5/2025	Class 3–4	Below 40%	HiCM-188	Intramyocardial injection	Incidence of major serious adverse events	35–75	NCT06340048
Human embryonic stem cell-derived cardiomyocyte therapy for chronic ischemic left ventricular dysfunction (HECTOR)	Cell	Recruiting	18	10/2025	Class 2–3	Below 40%	ESC-CMs	Cardiac catheter	Maximum tolerated dose	21–80	NCT05068674
Safety and efficacy of induced pluripotent stem cell-derived engineered human myocardium as biological ventricular assist tissue in terminal heart failure (BioVAT-HF)	Patch	Recruiting	53	10/2024	Class 3–4	Below 35%	hiPSC-CM	Left lateral mini thoracotomy or open chest surgery	Target heart wall thickness, heart wall thickening fraction	18–80	NCT04396899
A study of iPS cell-derived cardiomyocyte spheroids (HS-001) in patients with heart failure (LAPiS study) (LAPiS)	Spheroids	Recruiting	10	1/31/2026	Class 2+	Below 40%	HS-001 CS	Epicardial injection	Safety and tolerability	20–80	NCT04945018
Clinical trial of human (allogeneic) iPS cell-derived cardiomyocyte sheets for ischemic cardiomyopathy	Cell sheet patch	Closed	10	5/30/2024	Class 3–4	Below 35%	hiPSC-CM	Thoracotomy	Increased left ventricular ejection fraction compared preoperative levels	20+	jRCT2053190081

Table of current clinical trials.

### Intramyocardial injection trials

Allogenic hPSC-CMs have been delivered via intramyocardial injection during scheduled coronary artery bypass grafting (CABG) in two trials: one targeting patients with worsening ischemic heart disease (NCT05566600) and another enrolling patients with severe ischemic heart disease (NCT06340048). The HECTOR trial (NCT05068674) is evaluating transendocardial delivery of hPSC-CMs through cardiac catheterization.

The LAPiS trial (NCT04945018) administers allogeneic hPSC-CM spheroids intramyocardially to patients with severe HF. Five patients have been recruited into both a low dose and high dose cohort, 50 and 150 million hPSC-CMs, respectively, with early reports showing improved left ventricular ejection fraction (LVEF), decreased New York Heart Association (NYHA) classification, and reduced levels of N-terminal pro-B-type natriuretic peptide (NT-proBNP) (https://heartseed.jp/en/news/assets/2023/07/aa5fdd0940390720c758960ae066298f1c35d66c.pdf) (https://heartseed.jp/en/news/assets/2023/09/230911-Press%20Release-Heartseed_LAPiS_JCCvF.pdf).

### Epicardial patch trials

The BioVAT-HF trial (NCT04396899) employs epicardial patches composed of hPSC-CMs and stromal cells in a collagen 1 matrix to treat HF patients. Few adverse effects have been reported, and functional benefits at the maximal dose (800 M hPSC-CM/patient) include reductions in NYHA classification (from stage III to II) and increased ejection fraction^60^. Notably, the graft remained detectable after 3 months post-transplantation in one patient later undergoing heart transplantation^46^.

Another ongoing clinical trial in Japan (jRCT2053190081) uses hPSC-CM cell sheets^61,62^. Similar to BioVAT-HF trial, few adverse events were causally linked to treatment. Published results from three patients indicate functional recovery in two cases, with increased LVEF and decreased left ventricular end systolic and diastolic diameters observed at both 6 months and 1 year post-treatment^62^.

## Mechanism of repair

Engrafting hPSC-CMs into the myocardium to remuscularize damaged hearts was initially assumed to be the primary mechanism of cardiac repair. Murry’s group first demonstrated that hPSC-CMs functionally integrated into infarcted hearts, forming gap junctions with host cardiomyocytes via connexin-43^56^. Optogenetic silencing experiments confirmed this contribution, as contractile benefits were immediately lost when grafts were inhibited^63^.

Paracrine signaling has increasingly been recognized as another key mechanism of hPSC-CM mediated cardiac repair^64–66^. In particular, extracellular vesicles (EVs), including exosomes, have emerged potent mediators of cardiac recovery. Exosomes deliver bioactive cargos, proteins, messenger RNAs (mRNAs), microRNAs (miRNAs) and bioactive lipids, that modulate intercellular signaling^67–70^. In porcine MI models, EV injections improved cardiac function to a degree comparable with transplanted cells, underscoring their critical role in cardiac repair^66^.

Karbassi et al. further dissected this mechanism by generating noncontractile hPSC-CMs by knocking out slow skeletal TNNI1 and cardiac TNNI3, key components of the contractile machinery of hPSC-CMs^65^. Remarkably, these noncontractile hPSC-CMs preserved heart function after IR injury to a similar extent as wild-type hPSC-CMs, highlighting the importance of paracrine effects.

Both remuscularization and paracrine effects act in parallel. From a translational perspective, cell-free therapies may reduce risks associated with cell transplantation, but their rapid clearance may not confer long-term improvements to heart function as would a direct cell replacement therapy. By contrast, hPSC-CMs can act as a “living drug” after transplantation, providing contractile force, secreting pro-survival signals, and dynamically responding to host injury^71^.

## Challenges and mitigation strategies

### hPSC-CM population heterogeneity

**Fig. 3:**
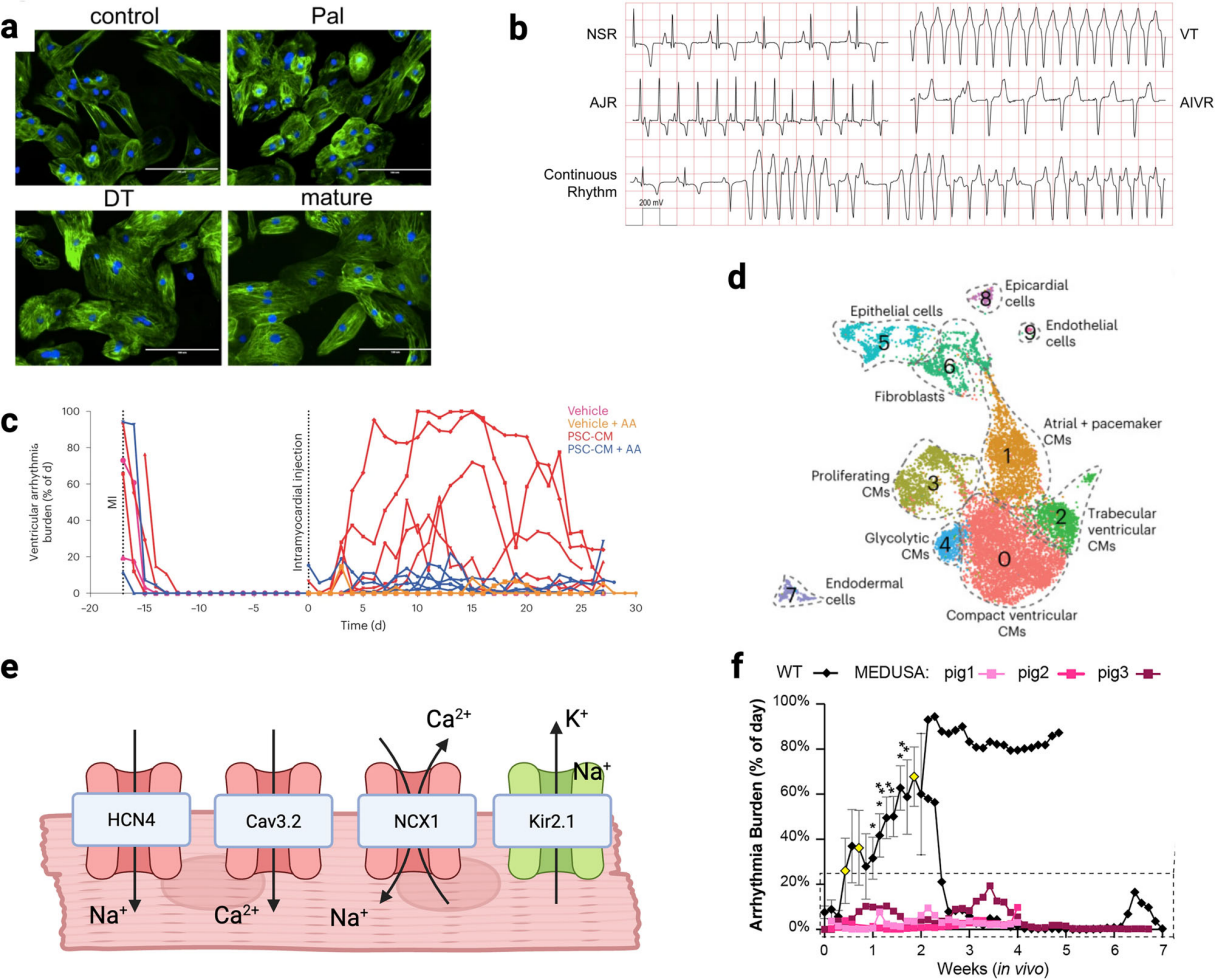
Mitigation strategies developed to increase intramyocardial injection therapeutic effectiveness. **a** Morphological differences in hPSC-CMs as they progressively matured with palmitate (Pal), Dex and T3 (DT) or PPar agonist + palmitate + Dex and T3 (mature)^23^. **b** ECG traces of arrhythmias present in a minipig injury model NSR normal, VT ventricular tachycardia, AJR accelerated junctional rhythm, AIVR accelerated idioventricular^82^. **c** Anti-arrhythmia drugs decrease the arrhythmogenic burden in hPSC-CM treated pigs^22^. **d** scRNAseq data of hPSC-CMs showing the multiple number of subpopulations contained within one culture^22^. **e** Diagram of gene edits made in MEDUSA hPSC-CMs to reduce automaticity. Red channels are knockouts, and green channels were knocked in. **f** Arrhythmogenic burden was decreased in the gene edited hPSC-CM treatment group^81^. **a** Modified from ref. ^23^ originally published under CC-BY 4.0 https://creativecommons.org/licenses/by/4.0/. **b** Modified from ref. ^82^ originally published under CC-BY 4.0 https://creativecommons.org/licenses/by/4.0/. **c**, **d** Modified from ref. ^22^ originally published under CC-BY 4.0. **f** Reprinted from ref. ^81^ with permission from Elsevier.

Single cell sequencing studies have highlighted the heterogeneous nature of hPSC-CM cultures^22^ (Fig. 3d). Thorough characterization of hPSC-CMs is critical to ensure reproducible therapeutic efficacy and to prevent aberrant in vivo differentiation of residual stem cells^40^. Atrial, ventricular, and nodal hPSC-CMs differ in electrophysiology, contractile function, and gene expression^22,72–75^. One study reported that populations with increased atrial and pacemaker-like cells led to increased rates of EA, with all animals demonstrating nearly sustained arrhythmia by day 8 post-transplantation^22^. The mechanisms underlying this increased arrhythmogenicity has not been fully explored. By contrast, ventricular specific hPSC-CMs have been differentiated^23,25,26^ and transplanted^23^, but their arrhythmia risk has not yet been investigated. A deeper mechanistic understanding of how hPSC-CM subtypes contribute to efficacy and safety is needed, and the optimal cell population for therapy remains undefined.

### hPSC-CM maturity

Compared to adult cardiomyocytes, hPSC-CMs display an immature phenotype characterized by differences in 1) morphology, 2) electrophysiology, 3) calcium handling, 4) contractility, 5) metabolism, and 6) proliferative capacity^76^. Immature hPSC-CMs are typically round, exhibit depolarized resting potentials, reliance on glycolysis, generate low contractile force, and lack T-tubules.

Most transplantation studies have used hPSC-CMs with an age of two to three weeks post-differentiation in vitro (Table 1). This immaturity may confer advantages, including increased proliferation and enhanced ischemic tolerances. However, the automaticity of hPSC-CMs has been implicated in EAs due to interference with host action potential propagation^76^. Interestingly, there is evidence of in situ maturation, with spontaneous resolution of EAs observed around 30 days after hPSC-CM transplantation^33,35,40^. It has been demonstrated that in vitro matured hPSC-CMs form grafts with improved structure and function in injured hearts^31^.

Numerous strategies have been explored to promote hPSC-CM maturity prior to transplantation. Biophysical approaches include modifying culture substrates, electrical pacing (1–2 Hz), and mechanical stretch have been explored^76,77^. Modifying culture substrates has been shown to have an effect on maturity: aligned fibers led to a more mature cell population at earlier time points^78^. In addition, substrates coated with PDMS of ~400 kPa stiffness increased hPSC-CM maturation^31^. When transplanted into cryoinjured guinea pigs, PDMS-cultured hPSC-CMs exhibited decreased arrhythmic burden relative to tissue culture plastic cultured hPSC-CMs.

In parallel, biochemical approaches such as supplementing fatty acids with hormones and peroxisome proliferator-activated nuclear receptor agonists to culture media have also been used to shift metabolism from glycolysis to fatty acid oxidation, yielding hPSC-CMs with a compact ventricular phenotype (Fig. 3a)^23^. Although these matured grafts were smaller, they contained more structurally and functionally mature hPSC-CMs.

Notably, the ideal maturation state for hPSC-CMs remains undefined. Optimal cells should maximize graft quality, such as increased contractility and calcium handling, while minimizing risks such as the enhanced arrhythmogenicity and reduced survival or proliferation. Determining this balance represents one of the field’s greatest challenges and opportunities.

### Cell survival and engraftment

Ensuring robust survival and integration of transplanted hPSC-CMs is essential for achieving lasting therapeutic benefits in patients. After transplantation, hPSC-CMs face multiple stressors including; hypoxia, oxidative stress, inflammation, and mechanical washout^48,56,79^. To address these challenges, Laflame et al. developed a pro-survival cocktail containing components that mitigate anoikis, apoptosis, necrotic, and mitochondrial cell death, while also enhancing ischemic tolerance^56^. This cocktail has enabled robust hPSC-CM engraftment in rodent and NHP models. In addition, microtissues such as spheroids have been employed to overcome mechanical washout and promote hPSC-CM engraftments^10^. Incorporation of electrically conductive silicon nanowires has been used to increase microtissue integration and enhanced functional recovery of host myocardium^9^.

Epicardial patches often suffer from low survival without sufficient vascularization. The addition of vascular supporting cells, particularly endothelial cells, has been used in patches to promote engraftment and vascular recruitment^48^. Other supporting vascular cells such as SMCs, have been shown to improve patches vascularization and functional recovery in pig models^8^. Beyond cellular composition, modulation of biochemical and physical cues within epicardial patches offers a powerful strategy to improve survival and engraftment. For example, Fan et al. engineered hPSC-CM patches to incorporate nanoparticles loaded with CHIR99021 and FGF147^80^ (Fig. 2g). These patches increased engraftment fourfold and stimulated hPSC-CM proliferation in a mouse model.

### Engraftment arrhythmia

Large animal studies have reported EAs that occur shortly after hPSC-CM injections, which typically resolve spontaneously after 1 month (Fig. 3b, c)^22,52,81^. EAs are thought to arise from the heterogenicity and immaturity of transplanted hPSC-CMs. Compared with intramyocardial injections, fewer EAs have been observed following transplantation of epicardial hPSC-CM patches, likely due to their physical insulation from host myocardium^50^. However, this same limited electrical integration can lead to unsynchronized contractions of transplanted patches and suboptimal therapeutic benefit^39^.

A straightforward and clinically translatable strategy for reducing arhythmic burden after hPSC-CM transplantation is the use of anti-arrhythmia drugs. Nakamura et al. screened several clinically relevant antiarrhythmic drugs and identified two effective options: amiodarone, a class III antiarrhythmic drug primarily a potassium channel inhibitor, and ivabradine, a HCN4 channel antagonist^82^. Amiodarone was delivered continuously, while ivabradine was administered during sustained tachycardia when porcine subjects reached heart rates greater than 150 bpm. This regimen eliminated fatal arrhythmias and reduced overall arrhythmic burden. Selvakumar et al. further confirmed the effectiveness of combined ivabradine and amiodarone, demonstrating decreased arrhythmia duration (in hours per day) and frequency (days with arrhythmia)^22^ (Fig. 3c). In addition, they evaluated catheter ablation, successfully mapping EAs to hPSC-CM injection sites, and decreasing the arrhythmogenic burden following catheter ablation. Notably, one porcine subject treated with hPSC-CMs composed of a higher atrial subpopulation, had recurrent EAs traced to secondary locations beyond the original ablation target.

To further probe EA mechanisms, Marchiano et al. conducted systematic genome editing to reduce hPSC-CMs automaticity^81^. By knocking out HCN4, Cav3.2, and NCX1, while overexpressing of Kir2.1, they generated cells capable of responding to action potentials without spontaneous firing. When transplanted into porcine models, these engineered hPSC-CMs substantially reduced arrhythmic burden (Fig. 3e, f).

### Immune rejection

Immune rejection remains a critical barrier to achieving long term hPSC-CM engraftment. While immunocompromised rodents (e.g., athymic rats) are widely used for transplantation studies, immunosuppression is currently the only viable option for large-animal and human trials.

Autologous hPSC-CMs are impractical for clinical use in acute MI treatment due to the 3–6 months required for manufacturing and their high cost^83,84^. In contrast, allogenic hPSC-CMs are more readily available and are being tested in all current clinical trials. However, they require either immunosuppressive regimens or human leukocyte antigen (HLA) matching to avoid rejection. Immunosuppressive drugs carry significant risks, particularly in vulnerable HF patients^85,86^. HLA class matching is a feasible alternative in relatively genetically homogenous populations such as Japan, where as few as 140 cell lines could match 90% of individuals^87^. By contrast, in genetically diverse populations such as United States, substantially larger HLA-matched cell banks are needed to achieve broad coverage, particularly for underrepresented ethnic groups^88^.

Emerging hypoimmune technologies offer a promising strategy to overcome these challenges. By genetically editing hPSC-CMs to eliminate expression of HLA class I and/or II molecules, these cells evade CD8^+^ and/or CD4^+^ T cell mediated killing^83^. To prevent natural killer (NK) cell mediated lysis, immune evasive factors such as CD47, and HLA-E/G can be knocked in^89^. Notably, hypoimmune gene-edited hPSC cardiac organoids have demonstrated the ability to restore contractile function in infarcted rat hearts and to improve graft retention and immune evasion in humanized mice relative to wild-type controls^90^.

## Conclusions and future perspectives

Over the past decade, hPSC-CM therapies advanced significantly, culminating in ongoing clinical trials. Progress in hPSC-CM differentiation and purification has enabled the production of clinical-grade hPSC-CMs, while intramyocardial injections and epicardial patches have emerged as promising delivery strategies. Early clinical trial results suggest these approaches improve cardiac function.

Despite this progress, several key challenges must be addressed before hPSC-CM therapies transform MI and HF treatment. Major obstacles include limited cell survival, low engraftment efficiency, and the risk of EAs. Pro-survival cocktails and co-transplantation with supporting cells have shown promise in enhancing hPSC-CM survival, but the optimal cell type and composition to maximize engraftment remains undefined. To mitigate EAs, anti-arrhythmic drugs, catheter ablation, and ion channels gene editing have been explored. While anti-arrhythmic drugs and catheter ablation are clinically feasible, further investigation is needed to minimize EA risk.

Optimizing the composition and maturity of transplanted hPSC-CMs is another critical challenge. Evidence suggests that hPSC-CM populations enriched in atrial-like subpopulations may increase arrhythmogenic risk, whereas whether ventricular-specific populations reduce EA remains undetermined. Striking the right balance between the proliferative and stress-tolerant properties of immature hPSC-CMs and the contractile and electrophysiological competence of mature hPSC-CMs will be essential to defining the ideal therapeutic cell product.

Immune rejection also remains a major barrier. Although current immunosuppression regimes are effective, they pose significant risks, highlighting the need for alternative approaches. Hypoimmune technologies, pioneered in hPSC-derived pancreatic islet transplantation, offer promising strategies for cardiac regenerative medicine.

Lastly, a deeper mechanistic understanding of hPSC-CM-mediated cardiac repair is essential. The relative contributions of remuscularization compared to paracrine effects remain incompletely understood, and resolving this will be critical for refining therapeutic strategies.

Looking ahead, integrating emerging innovations in cell engineering, immunomodulation, and tissue engineering will be key to overcoming these challenges and realizing the full potential of hPSC-CM therapies for treatment of MI and HF.
